# Case report: Genotype and phenotype of *DYNC1H1*-related malformations of cortical development: a case report and literature review

**DOI:** 10.3389/fneur.2023.1163803

**Published:** 2023-04-25

**Authors:** Wen-Rong Ge, Pei-Pei Fu, Wei-Na Zhang, Bo Zhang, Ying-Xue Ding, Guang Yang

**Affiliations:** ^1^Department of Pediatrics, Beijing Friendship Hospital, Capital Medical University, Beijing, China; ^2^Department of Neurology and ICCTR Biostatistics and Research Design Center, Boston Children's Hospital, Harvard Medical School, Boston, MA, United States; ^3^Senior Department of Pediatrics, The Seventh Medical Center of People's Liberation Army General Hospital, Beijing, China; ^4^Department of Pediatrics, The First Medical Center, Chinese People's Liberation Army General Hospital, Beijing, China; ^5^The Second School of Clinical Medicine, Southern Medical University, Guangzhou, China

**Keywords:** DYNC1H1 gene, malformations of cortical development, variant, microtubule-binding domain, case report

## Abstract

**Background:**

Mutations in the dynein cytoplasmic 1 heavy chain 1 (*DYNC1H1*) gene are linked to malformations of cortical development (MCD), which may be accompanied by central nervous system (CNS) manifestations. Here, we present the case of a patient with MCD harboring a variant of *DYNC1H1* and review the relevant literature to explore genotype-phenotype relationships.

**Case presentation:**

A girl having infantile spasms, was unsuccessfully administered multiple antiseizure medications and developed drug-resistant epilepsy. Brain magnetic resonance imaging (MRI) at 14 months-of-age revealed pachygyria. At 4 years-of-age, the patient exhibited severe developmental delay and mental retardation. A *de novo* heterozygous mutation (p.Arg292Trp) in the *DYNC1H1* gene was identified. A search of multiple databases, including PubMed and Embase, using the search strategy *DYNC1H1* AND [malformations of cortical development OR seizure OR intellectual OR clinical symptoms] up to June 2022, identified 129 patients from 43 studies (including the case presented herein). A review of these cases showed that patients with *DYNC1H1*-related MCD had higher risks of epilepsy (odds ratio [OR] = 33.67, 95% confidence interval [CI] = 11.59, 97.84) and intellectual disability/developmental delay (OR = 52.64, 95% CI = 16.27, 170.38). Patients with the variants in the regions encoding the protein stalk or microtubule-binding domain had the most prevalence of MCD (95%).

**Conclusion:**

MCD, particularly pachygyria, is a common neurodevelopmental disorder in patients with *DYNC1H1* mutations. Literature searches reveales that most (95%) patients who carried mutations in the protein stalk or microtubule binding domains exhibited DYNC1H1-related MCD, whereas almost two-thirds of patients (63%) who carried mutations in the tail domain did not display MCD. Patients with *DYNC1H1* mutations may experience central nervous system (CNS) manifestations due to MCD.

## Introduction

The dynein cytoplasmic 1 heavy chain 1 (*DYNC1H1*) gene encodes a large (530 kDa) component of the multisubunit dynein motor complex that mediates retrograde axonal transport and other intracellular processes. These processes include Golgi apparatus regulation, cargo transport, axonal transport, spindle pole organization, organelle motility, and nuclear migration throughout mitosis (1, 2). *DYNC1H1* mutations are associated with various clinical manifestations that are typically divided into two major categories: central nervous system (CNS) symptoms (e.g., malformations of cortical development [MCD], intellectual developmental disorder, and developmental and epileptic encephalopathy [DEE]) (3–8); and neuromuscular diseases (e.g., spinal muscular atrophy [SMA] with predominance in the lower extremities and Charcot–Marie–Tooth [CMT], type 20 (1, 2, 9, 10).

The *DYNC1H1* protein has four domains: the tail (amino acid [aa] residues 1–1373 and 4222–4646); the ATP-binding AAA motor domain (residues 1868–3168 and 3553–4221); the linker (residues 1374–1867); and the stalk or microtubule-binding domain (MBD; residues 3169–3552) (8). With a gene constraint Z score of 10.97 in the Genome Aggregation Database(gnomAD), *DYNC1H1* is known to be exceptionally susceptible to missense mutations (11), which account for most of the *DYNC1H1* variants identified to date (11). It remains unclear as to whether any association exists between the location of mutations in the *DYNC1H1* gene and the complex and variable clinical symptoms observed in individuals harboring these variants.

MCDs are structural abnormalities that impede normal cortical development, including microcephaly, macrocephaly, brain overgrowth spectrum, focal cortical dysplasia, grey matter heterotopia, lissencephaly, cobblestone malformation, polymicrogyria, radiological spectrum of cobblestone malformation and polymicrogyria, schizencephaly, and dysgyria (12). Patients with MCDs may be asymptomatic or exhibit a range of symptoms, including cognitive impairment, developmental delay, and epilepsy with drug resistance (13). MCD is a common(28%) condition associated with *DYNC1H1* mutations (14). However, the association between MCD and clinical symptoms—especially those affecting the CNS—has yet to be comprehensively evaluated in individuals with *DYNC1H1* mutations.

In this study, we present the case of a child with MCD owing to a missense mutation in the *DYNC1H1* gene and review the literature to explore the association between the location of gene variants and MCD or other clinical symptoms.

## Case presentation

**Figure 1:**
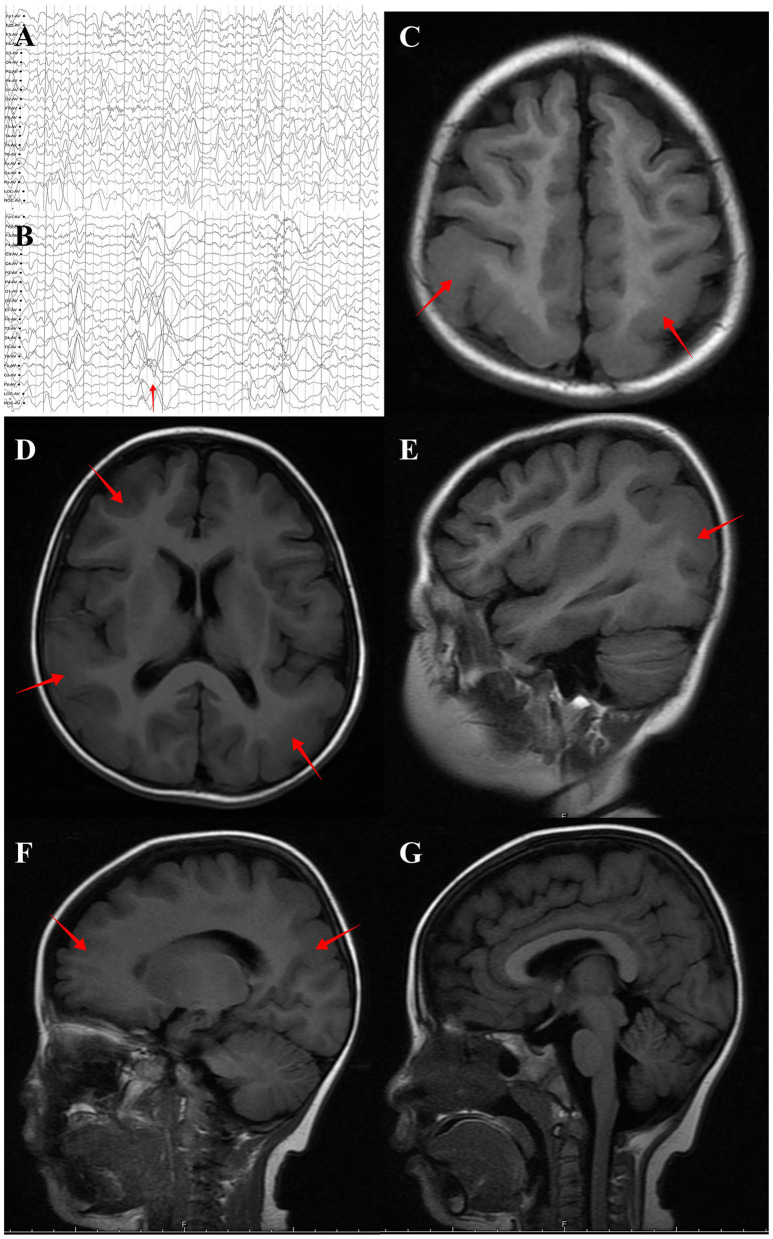
Brain MRI and EEG of the individual with *DYNC1H1* variant p.Arg292Trp. **(A)** Interictal EEG showed multifocal spike-wave complexes at 2 years old. **(B)** EEG at spasm onset (2 years old) revealed brief, high γ oscillations combined with slow delta waves (red arrow). **(C)** (Axial plane, T1 flair), **(D)** (Axial plane, T1 flair), (**E)** (Sagittal plane, T1 flair), **(F)** (Sagittal plane, T1 flair) and **(G)** (Sagittal plane, midline, T1 flair). MRI performed at 4 years old and revealed posterior predominant pachygyria (red arrow).

The case was a 4-year-old girl and is the only child of unrelated and healthy parents without a family history of neurological disorders. The patient was born at term by spontaneous delivery and exhibited no postnatal abnormalities. The onset of seizures as epileptic spasms began at the age of 5 months. At this time, electroencephalography (EEG) revealed hypsarrhythmia, thus leading to a diagnosis of infantile spasms. Adrenocorticotropic hormone and multiple antiseizure medications, including vigabatrin, failed to control the seizures. A ketogenic diet and vagus nerve stimulation were ineffective at treating the condition, and spasms began when the child was 2 years-of-age (Figures 1A, B). Over time, the type of seizure evolved into absence seizures (confirmed by EEG). The patient exhibits severe developmental delay, with independent sitting at 9 months, and remains unable to walk unassisted. These symptoms were accompanied by severe intellectual disabilities and poor language skills, with a vocabulary consisting of only 10 words.

**Figure 2:**
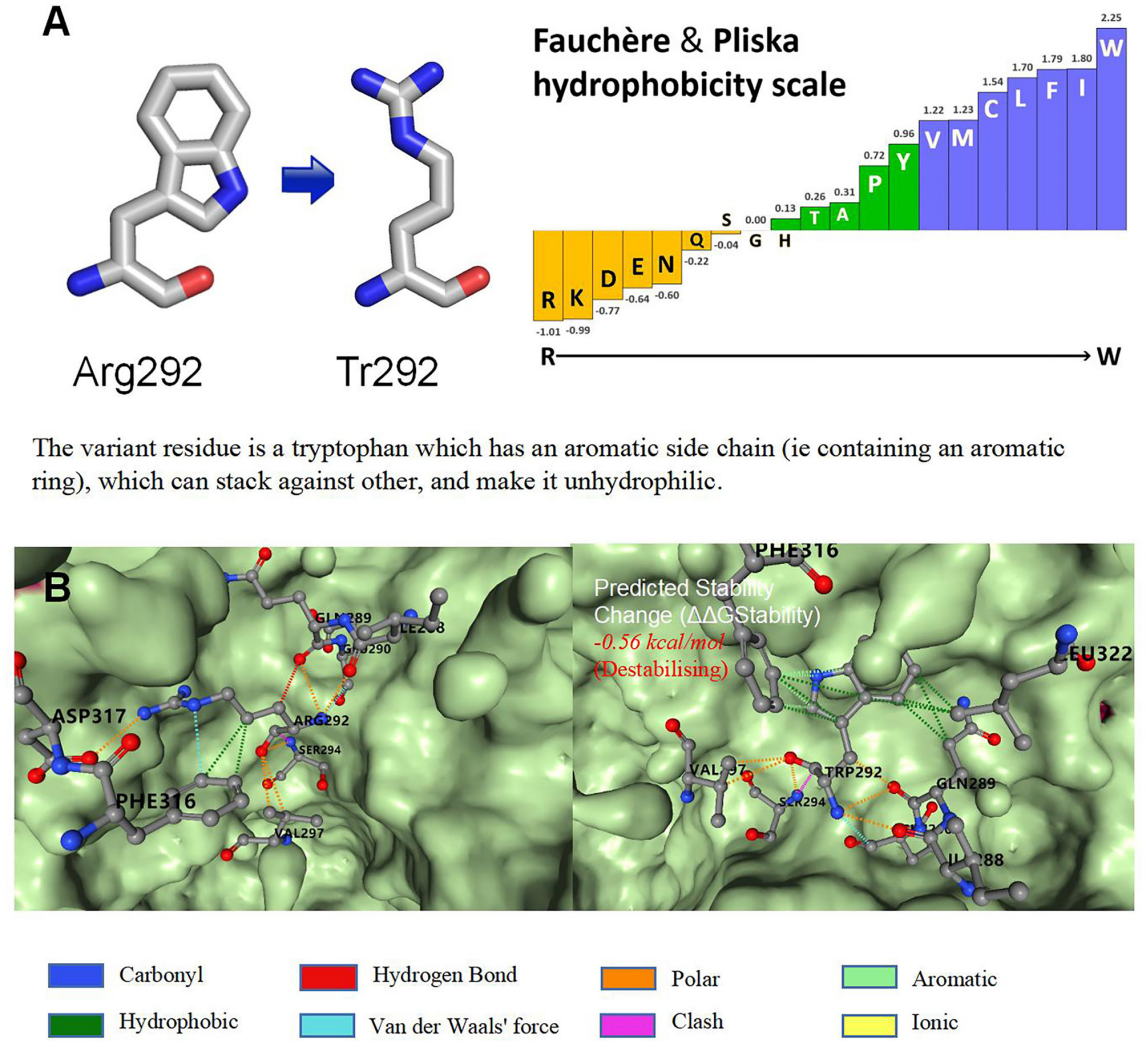
Function and stability prediction of variants. **(A)** Prediction of p.Arg292Trp function. **(B)** Prediction of variant stability.

Brain magnetic resonance imaging (MRI) revealed small, broad, and flat gyri in the bilateral multiple lobes, indicating pachygyria (Figures 1C–F). Genomic DNA of the proband and her parents was extracted from peripheral blood for trio-whole-exome-sequencing (WES), using a method similar to that used in our previous study (15).The results revealed a *de novo* heterozygous variant (p.Arg292Trp) in the *DYNC1H1* gene. In *silico* analysis using PolyPhen-2 and MutationTaster further indicated that the p.Arg292Trp mutation was “probably damaging,” which was consistent with the PROVEAN server data; the variant was categorized as probably pathogenic according to the American College of Medical Genetics and Genomics guidelines (Supplementary Figure S1). Function- and stability-change predictions were performed as previously described (16, 17). The results showed that this variant caused significant damage to the encoded protein (Figure 2).

## Genotype–phenotype analysis

Relevant studies were identified from the existing literature by two independent reviewers (WG and PF, two pediatric neurologists with more than 10 years of experience) by searching MEDLINE, PubMed, Embase, Google, and the China National Knowledge Infrastructure. Most studies on DYNC1H1 have found that most mutations are missense mutations, while mutations that clearly result in protein truncation, such as frameshift or nonsense mutations, are relatively rare (14). At present, the pathogenic mechanism of DYNC1H1 is not fully understood. Animal study has shown that heterozygosity for a null mutation in DYNC1H1 does not cause overt phenotypes in mice (18). In light of this, we only included patients carrying missense mutations in the present study. The search strategy utilized was as follows: DYNC1H1 AND/OR missense AND [malformations of cortical development OR seizure OR intellectual OR clinical symptoms] until June 2022. The search results were restricted to studies in humans that were published in either English or Chinese. To ensure an accurate diagnosis of MCD, we excluded subjects without brain MRI reports.

SPSS v26.0 (IBM, Armonk, NY, USA) and Prism (GraphPad, La Jolla, CA, USA) software were used for statistical analysis. Data were analyzed with the chi-squared test. A logistic regression model was used to identify risk factors for MCD. Differences with *p* < 0.05 were considered statistically significant.

**Figure 3:**
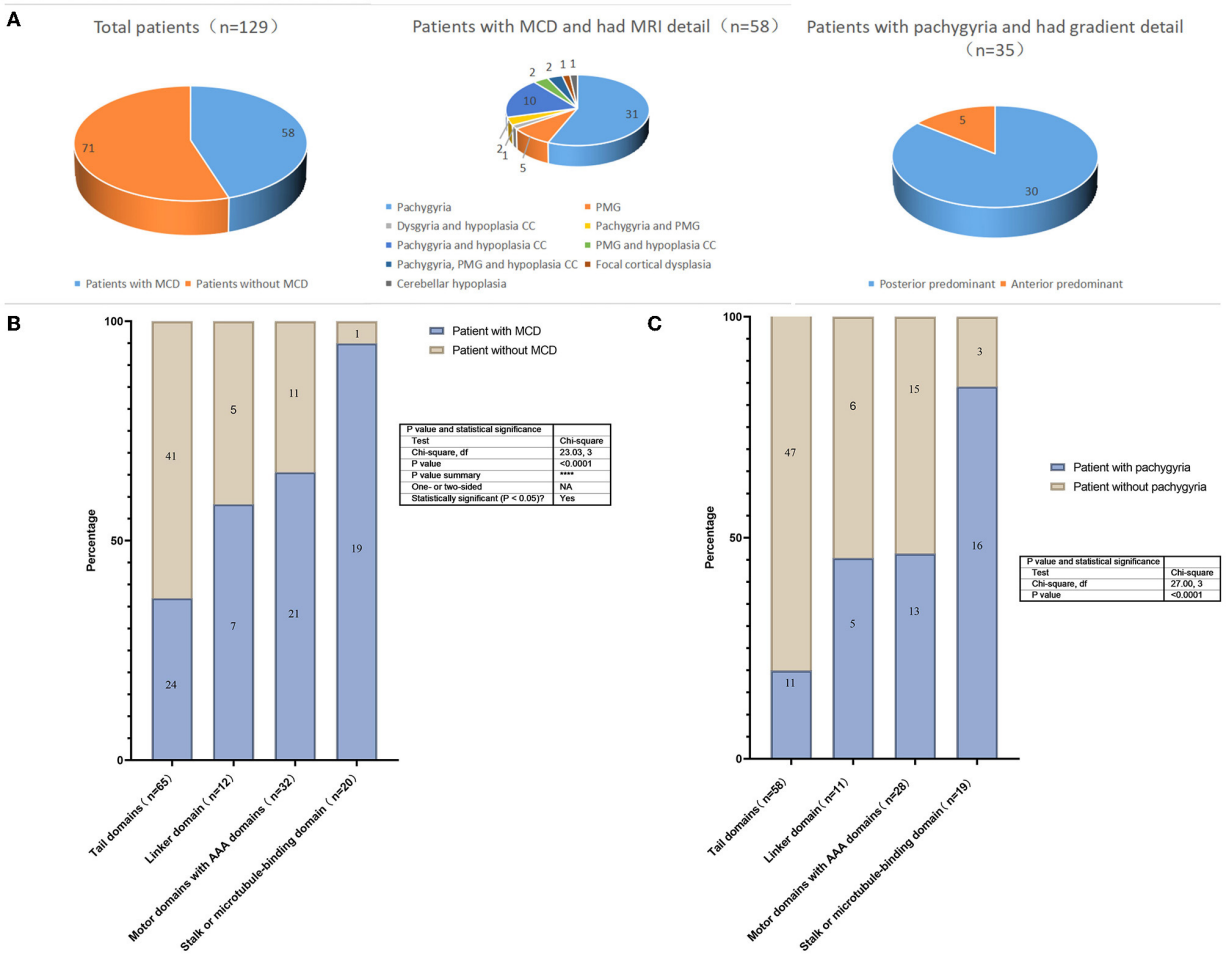
**(A)** The proportion of malformations of cortical development and subtype in the patients with *DYNC1H1* mutations; **(B)** Prevalence of MCD. Prevalence of different variants in the 4 domains of the *DYNC1H1* protein; **(C)** Prevalence of pachygyria. Prevalence of different variants in the 4 domains of the *DYNC1H1* protein. PMG, polymicrogyria; CC, corpus callosum.

In total, we identified 129 cases (including the patient reported in the current study) with *DYNC1H1* missense variants and neurologic/developmental phenotypes from 43 studies (3–5, 8–10, 14, 19–54). Among them, there were 71 individuals with MCD (55%), 81 (63%) with intellectual disability/developmental delay (ID/DD), and 59 (46%) with epilepsy (Supplementary Table S1; Figure 3A). The proportion of patients with epilepsy was higher in the MCD group than among those without MCD; the same was true for patients with vs. without ID/DD (Table 1). MCD was associated with an elevated risk of epilepsy (odds ratio [OR] = 33.67, 95% confidence interval [CI] = 11.59, 97.84) or ID/DD (OR = 52.64, 95% CI = 16.27, 170.38) (Table 1). In these MCD patients, 58 cases reported the details of their MRI, 45 had pachygyria(one as agyria)(45/58, 78%), and gradient details were available for 35 patients; most of them (30/35, 86%) were posterior predominant, while the remaining five patients were anterior predominant. Of the 45 patients with pachygyria, 41 had epilepsy(41/45, 91%). In addition, 15 patients had hypoplasia of the corpus callosum(15/58, 26%), 11 had polymicrogyria(11/58, 19%) and seven among them were frontal (7/11, 64%) (Supplementary Table S1; Figure 3A). Of the 51 patients for which the time of seizure onset was reported, 37 had an onset in infancy(37/51, 73%). Of the 22 patients with known seizure outcomes, 18 developed drug-resistant epilepsy(18/22, 82%). Patients with pachygyria had a higher proportion of epilepsy and ID/DD when compared to those without MCD (Table 1). Pachygyria was associated with an elevated risk of epilepsy (odds ratio [OR] = 108.65, 95% confidence interval [CI] = 27.43, 430.38) or ID/DD (OR = 44.00, 95% CI = 11.79, 164.17) (Table 1). Compared with patients with other subtype of MCD(n=13), patients with pachygyria had a higher proportion and an elevated risk of epilepsy(OR = 11.96, 95% CI = 2.67, 53.47) (Table 1).

**Table 1:** Comparison of central nervous system disorders in patients with *DYNC1H1* variants with or without malformations of cortical development and with or without pachygyria.

	**Patients with MCD (*n* = 71)**	**Patients without MCD (*n* = 58)**	**χ^2^**	**OR**	**95% CI**	***P* value**
Epilepsy	54 (76%)	5 (9%)	58.49	33.67	(11.59, 97.84)	< 0.001^a^
						< 0.001^b^
ID/DD	67 (94%)	14 (24%)	67.39	52.64	(16.27, 170.38)	< 0.001^a^
						< 0.001^b^
	Patients with pachygyria (*n* = 45)	Patients with MCD but without pachygyria (*n* = 13)				
Epilepsy	41 (91%)	6 (46%)	10.51	11.96	(2.67, 53.47)	0.001^a^
						0.001^b^
ID/DD	42 (93%)	12 (92%)		1.167	(0.11, 12.26)	1.000^c^
						0.898^b^
	Patients with pachygyria (*n* = 45)	Patients without MCD (*n* = 58)				
Epilepsy	41 (91%)	5 (9%)	69.77	108.65	(27.43, 430.38)	< 0.001^a^
						< 0.001^b^
ID/DD	42 (93%)	14 (24%)		44.00	(11.79, 164.17)	< 0.001^c^
						< 0.001^b^

CI, confidence interval; DYNC1H1, cytoplasmic dynein 1 heavy chain 1; ID/DD, intellectual disability/developmental delay; MCD, malformations of cortical development; OR, odds ratio.

^a^Chi-squared test.

^b^Logistic regression analysis.

^c^Fishers' exact test.

There were 93 mutations identified in the 129 individuals: 44 mutations were located in the tail domain (44/93, 47%), 10 in the linker domain (10/93, 11%), 25 in the motor (AAA) domain (25/93, 27%), and 14 in the stalk/MBD(14/93, 15%). There were statistically significant differences in the prevalence of MCD prevalence according to the domain in which the *DYNC1H1* variant was located. The highest prevalence was observed in patients with variants in the stalk/MBD (95%; Figure 3B). The same phenomenon has also been reported in children with pachygyria in that the highest prevalence was observed in patients with variants in the stalk/MBD (84%, Figure 3C).

## Discussion

*DYNC1H1*-related disorders are heterogeneous and can affect the development and function of the CNS, peripheral nervous system (PNS), or both (14). Historically, more attention has been given to PNS diseases such as SMA or CMT than CNS manifestations. In this study, we reported a case of a *DYNC1H1* variant presenting with CNS symptoms, reviewed the literature on *DYNC1H1* mutations in MCD, and identified a higher prevalence of MCD than previously reported (14). We suspect that this discrepancy may be because we excluded subjects without brain MRI data. Although *DYNC1H1* is widely expressed in different tissues, our multi-databases searches (e.g., Genotype-Tissue Expression, Human Protein Atlas, Functional Annotation of the Mammalian Genome, and ConsensusPathDB) indicated that *DYNC1H1* expression may be higher in brain and muscle tissues, especially in the cerebral cortex and skeletal muscles. The DYNC1H1 protein is also involved in neuron-specific processes, specifically retrograde axonal transport. Cytoplasmic DYNC1H1 is an essential component of the cytoplasmic dynamic protein complex and is upregulated during normal development of the nervous system (2, 55). In a previous study, Hoang et al. suspected that the mutations found in *DYNC1H1* and associated with disease may have either a dominant-negative or dominant gain-of-function effect (56). Their study showed that these mutations strongly inhibited the gliding of microtubules by dyneins immobilized on surfaces, compromised the activation of processive dynein movement, reduce the run length of processive dynein-dynactin-N-terminal coiled-coil domain of Bicaudal-D2 complexes, and ultimately compromise the expression and motility of the dynein complex *in vitro* (56). Furthermore, Hoang et al. found that the mutations with the strongest effects on dynein motility were associated with MCD in humans (56). The mutation detected in our patient (p.Arg292Trp) was previously reported by Benson et al. (43), and a previous study reported that a female with this mutation had focal epilepsy (with onset between 13–18 months) and intellectual disability. A similar phenotype was observed in our patient in that she had early-onset epilepsy and severe intellectual disability.

Lissencephaly is a subtype of MCD that is characterized by a thickened cortex and a gyral abnormality ranging from agyria to pachygyria. This condition usually involves the entire brain or large areas of the cerebral hemispheres with an anterior or posterior predominance (12). Many genes have been identified as being associated with lissencephaly, including *LIS1, DCX, ARX, CDK5*, and *DYNC1H1*. Various genes may cause the differences in gradients observed. *LIS1*-related lissencephaly is more likely to result in a posterior-to-anterior gradient, whereas *DCX*-related lissencephaly is associated with an anterior-to-posterior gradient (57). For *DYNC1H1*-related MCDs, Scoto et al. (10) demonstrated posterior predominant lesions were the most common manifestations. Our current analysis showed that the most common subtype of *DYNC1H1*-related MCD was posterior predominant pachygyria, this finding was consistent with the conclusions drawn by Scoto et al. (10). Another subtype, polymicrogyria, is unlike pachygyria in that iappears to be more predominant in the frontal region. MCDs have been previously linked to hypoplasia of the corpus callosum. In this study, we identified 15 MCD patients with corpus callosum hypoplasia; consequently, we consider that this may be one of the characteristic features of *DYNC1H1*-related MCD.

Besides imaging abnormalities, CNS symptoms have been observed in patients with MCD and the *DYNC1H1* mutations. Most individuals with MCD have epilepsy and/or ID/DD. Our literature review found that 76% of individuals with MCDs had recurrent seizures, which is consistent with the rate of 75% reported in a previous study (58). MCDs are an important cause of epilepsy, especially early-stage medically refractory epilepsy (59). In this study, cases in which where clinical details were available indicated that epilepsy had an early onset, and the patient subsequently developed drug resistance. Seizures occur in over 90% of children with lissencephaly (57). Compared to other MCD subtypes with vary widely clinical manifestations, such as PMG and macrocephaly, children with lissencephaly have a relatively higher risk of developing epilepsy (57). In our study, 91% (41/45) of patients with pachygyria/agyria had epilepsy; in addition, the risk and proportions of occurrence were significantly increased when compared to children with other subtypes of MCD, this finding was consistent with a previous report (57). MCD patients may present with developmental delay and cognitive impairment (60); however, according to our literature review, the proportion of ID/DD cases among patients with MCD was alarmingly high. We believe that two factors contribute to this phenomenon: neurological dysfunction caused by the *DYNC1H1* mutation, and the cognitive impairment caused by seizure onset. It is important to consider that minor imaging abnormalities may also be involved and could have been overlooked in previous investigations. After comparing the symptoms of patients with and without MCD, we consider that CNS symptoms are more likely to be due to MCD caused by *DYNC1H1* variants than the mutation itself.

While the majority of individuals had mutations in the motor domain stalk, patients with mutations in the DYNC1H1 tail domain were more likely to have isolated neuromuscular symptoms such as SMA and CMT (52). Furthermore, patients with mutations in the motor domain (including the AAA domain and MBD or stalk) are more likely to develop MCD (19, 61), which is supported by the results of our literature review. In addition, we found that patients with variants in the stalk/MBD are more likely to develop pachygyria. However, given the large size of the DYNC1H1 protein, and its complex interactions with various other proteins, mutations that affect tertiary structure could also result in alterations to functional outcomes and symptomatology (54). Therefore, the results of this study should be interpreted with caution.

There were some limitations to the current study that need to be considered. First, the nature of the literature review did not allow us to acquire any additional information on the reported cases. Second, to ensure an accurate diagnosis of MCD, we excluded some patients with clinical symptoms but without brain MRI examination, thus reducing the reliability of our results. Third, due to the lack of detailed information for some cranial MRI results, particularly with regards to the gradient of pachygyria, it was not possible to determine the precise proportion of posterior predominant pachygyria in all patients with MCD, even though it is the most commonly observed manifestation, Moreover, the analysis results related to pachygyria must also be treated with caution. Although these findings are of interest at the theoretical level, they require experimental validation in future studies.

In conclusion, posterior predominant pachygyria (a subtype of MCD) is a common neurodevelopmental disorder in patients that possess *DYNC1H1* mutation. Most patients (95%) who carry mutations in the protein stalk or microtubule binding domains exhibit DYNC1H1-related MCD, whereas almost two-thirds (63%) who carry mutations in the tail domain do not display MCD. In patients with *DYNC1H1*-related disorders, CNS symptoms tend to be associated with MCD, with seizure onset often occurring in infancy and drug resistance developing over time.

## Data availability statement

The datasets presented in this article are not readily available because of ethical and privacy restrictions. Requests to access the datasets should be directed to the corresponding authors.

## Ethics statement

The studies involving human participants were reviewed and approved by the Ethics Committee of the First Medical Center of the People's Liberation Army General Hospital. Written informed consent to participate in this study was provided by the participants' legal guardian/next of kin. Written informed consent was obtained from the minor(s)' legal guardian/next of kin for the publication of any potentially identifiable images or data included in this article.

## Author contributions

Y-XD and GY participated in study conception and design. P-PF, W-RG, and BZ acquired the data. W-RG, W-NZ, and BZ analyzed the data. W-RG drafted the initial version of the manuscript. All authors participated in manuscript revision for intellectual content and approved the final published version.
