# Supplementary material for: Case report: Genotype and phenotype of DYNC1H1-related malformations of cortical development: a case report and literature review
Source: Front Neurol. 2023 Apr 25;14:1163803. doi: 10.3389/fneur.2023.1163803 (PMC10167015; doi:10.3389/fneur.2023.1163803)

Figure S1. Genetic testing results and pathogenicity according to ACMG guidelines.

Variant	PolyPhen-2	Mutation Taster	PHRED-like scaled CADD scores	SIFT	ACMG
DYNC1H1; chr14:102446800; c.874C>T,p.Arg292Trp	0.986(Probably damaging)	Disease causing	28.8	0.01(Deleterious)	Likely pathogenic(PS2+PP2+PP3+PP4)

Patient's forward Sanger sequencing.

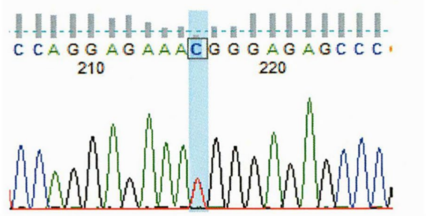

Patient's reverse Sanger sequencing.

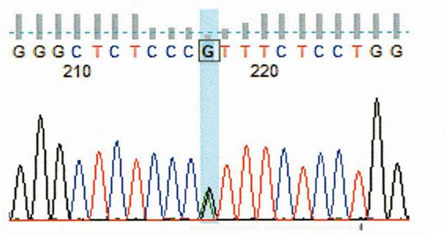

Patient's father's forward Sanger sequencing.

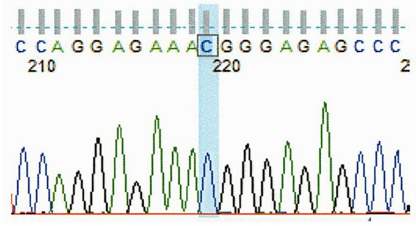

Patient's father's reverse Sanger sequencing.

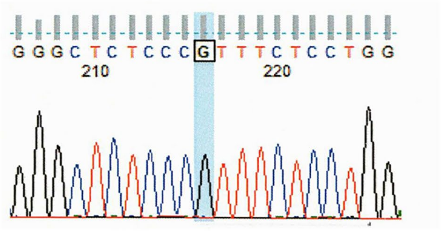

Patient's mother's forward Sanger sequencing.

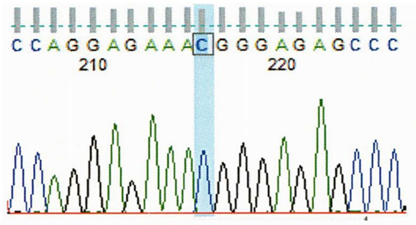

Patient's mother's forward sanger sequencing.

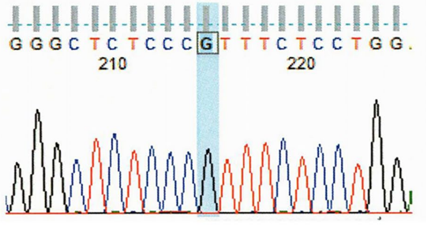

Supplement: Supplementary file 1 [file Image_1.pdf]
