# Supplementary material for: Case report: Genotype and phenotype of DYNC1H1-related malformations of cortical development: a case report and literature review
Source: Front Neurol. 2023 Apr 25;14:1163803. doi: 10.3389/fneur.2023.1163803 (PMC10167015; doi:10.3389/fneur.2023.1163803)
Supplement: Supplementary file 2 [file Table_1.DOCX]

| Table S1. 129 patients’variants and phenotype. AAA= ATP-binding AAA motor domain; CC= corpus callosum; CMT/HMN=Charcot-Marie Tooth/Hereditary Motor Neuropathy; ID/DD= intellectual disability/developmental delay; MCD= malformations of cortical development; N=No; NA= not apply; NR=not report; SMA= spinal muscular atrophy; PMG= polymicrogyria; Y=Yes. | | | | | | | | | | |
| --- | --- | --- | --- | --- | --- | --- | --- | --- | --- | --- |
| No. | Variants | Location | Seizure | Seizure onset at infancy | ID/DD | SMA | CMT/HMN | MRI abnormallities | MCD | MRI details |
| 1 | p.Lys129Ile | N-terminal | Y | N | Y | N | N | Y | Y | Posterior predominant pachygyria; hypoplasia CC |
| 2 | p.Gly192Arg | N-terminal | N | NA | N | Y | Y | N | N |  |
| 3 | p.Gln197Glu | N-terminal | N | NA | N | N | Y | N | N |  |
| 4 | p.Gln198Lys | N-terminal | N | NA | N | N | Y | N | N |  |
| 5 | p.Arg251Cys | N-terminal | N | NA | Y | Y | N | Y | N |  |
| 6 | p.Arg251Cys | N-terminal | N | NA | N | Y | N | Y | N |  |
| 7 | p.Arg251Cys | N-terminal | N | NA | N | Y | N | Y | N |  |
| 8 | p.Arg251Cys | N-terminal | N | NA | Y | Y | N | N | N |  |
| 9 | p.Arg251Leu | N-terminal | N | NA | N | Y | N | N | N |  |
| 10 | p.Arg251Cys | N-terminal | N | NA | Y | Y | N | Y | Y | NR |
| 11 | p.Arg251His | N-terminal | N | NA | N | N | Y | N | N |  |
| 12 | p.Arg264Gln | N-terminal | N | NA | Y | Y | N | Y | Y | NR |
| 13 | p.Arg264Gln | N-terminal | N | NA | N | Y | N | N | N |  |
| 14 | p.Arg264Leu | N-terminal | N | NA | N | Y | N | N | N |  |
| 15 | p.Arg292Gln | N-terminal | N | NA | N | N | Y | N | N |  |
| 16 | p.Arg292Trp | N-terminal | Y | Y | Y | N | N | Y | Y | Posterior predominant pachygyria |
| 17 | p.Arg292Trp | N-terminal | Y | Y | Y | N | N | Y | Y | Frontal cortical dysplasia |
| 18 | p.Lys305Asn | N-terminal | Y | Y | Y | N | N | Y | Y | Posterior predominant pachygyria |
| 19 | p.His306Arg | dimerization domain | N | NA | N | Y | N | N | N |  |
| 20 | p.His306Arg | dimerization domain | N | NA | N | Y | N | N | N |  |
| 21 | p.Arg309His | dimerization domain | Y | Y | Y | N | N | Y | Y | Posterior predominant pachygyria |
| 22 | p.Arg309His | dimerization domain | Y | Y | Y | N | N | Y | Y | Posterior predominant pachygyria/agyria; hypoplasia CC |
| 23 | p.Arg309His | dimerization domain | Y | Y | Y | N | N | Y | Y | Posterior predominant pachygyria |
| 24 | p.His311Pro | dimerization domain | Y | Y | Y | Y | N | Y | Y | NR |
| 25 | p.Asp338Asn | dimerization domain | N | NA | N | Y | N | N | N |  |
| 26 | p.Asp338Asn | dimerization domain | N | NA | N | N | Y | N | N |  |
| 27 | p.Arg399Gly | dimerization domain | N | NA | N | N | Y | N | N |  |
| 28 | p.Arg399Gly | dimerization domain | N | NA | N | N | Y | N | N |  |
| 29 | p.Leu420Arg | dimerization domain | Y | Y | Y | N | N | Y | Y | NR |
| 30 | p.Val560Met | Interaction with DYNC1I | N | NA | N | Y | N | N | N |  |
| 31 | p.Glu561Gly | Interaction with DYNC1I | Y | N | N | N | Y | Y | Y | Posterior predominant pachygyria； hypoplasia CC |
| 32 | p.Met581Leu | Interaction with DYNC1I | N | NA | N | Y | N | Y | N |  |
| 33 | p.Ile584Leu | Interaction with DYNC1I | N | NA | Y | Y | N | N | N |  |
| 34 | p.Ile584Leu | Interaction with DYNC1I | Y | Y | Y | Y | N | Y | N |  |
| 35 | p.Arg598Cys | Interaction with DYNC1I | N | NA | N | N | Y | N | N |  |
| 36 | p.Arg598Cys | Interaction with DYNC1I | N | NA | N | Y | N | N | N |  |
| 37 | p.Arg598Cys | Interaction with DYNC1I | N | NA | N | Y | N | N | N |  |
| 38 | p.Glu603Asp | Interaction with DYNC1I | N | NA | N | N | Y | N | N |  |
| 39 | p.Glu603Val | Interaction with DYNC1I | N | NA | N | Y | N | N | N |  |
| 40 | p.Val612Met | Interaction with DYNC1I | N | NA | N | Y | N | N | N |  |
| 41 | p.Val612Met | Interaction with DYNC1I | N | NA | N | Y | N | N | N |  |
| 42 | p.Val612Met | Interaction with DYNC1I | N | NA | N | Y | N | N | N |  |
| 43 | p.Val612Met | Interaction with DYNC1I | N | NA | N | Y | N | N | N |  |
| 44 | p.Val612Met | Interaction with DYNC1I | N | NA | Y | Y | N | N | N |  |
| 45 | p.Glu666Asp | Interaction with DYNC1I | Y | NR | Y | N | N | Y | Y | Posterior predominant pachygyria；heterotopia； hypoplasia brain stem |
| 46 | p.Val668Asp | Interaction with DYNC1I and DYNC1LI | Y | N | Y | N | N | Y | Y | Posterior predominant pachygryia |
| 47 | p.Leu774Pro | Interaction with DYNC1LI | N | NA | Y | N | N | Y | Y | Posterior predominant pachygryia；large corpus callosum |
| 48 | p.Pro776Leu | Interaction with DYNC1LI | N | NA | N | Y | N | N | N |  |
| 49 | p.Gly807Ser | dimerization domain | N | NA | N | Y | N | N | N |  |
| 50 | p.Val823Leu | dimerization domain | N | NA | Y | N | N | Y | Y | NR |
| 51 | p.Tyr970Cys | dimerization domain | N | NA | Y | Y | N | Y | Y | Hypoplasia CC;Frontal PMG |
| 52 | p.Tyr970Cys | dimerization domain | N | NA | Y | Y | N | Y | Y | Frontal PMG |
| 53 | p.Tyr970Cys | dimerization domain | N | NA | N | Y | N | Y | Y | Frontal PMG |
| 54 | p.Thr1013Ile | dimerization domain | N | NA | N | N | Y | N | N |  |
| 55 | p.Phe1093Ser | dimerization domain | Y | Y | Y | N | N | N | N |  |
| 56 | p.Asp1062Gly | dimerization domain | Y | N | Y | N | N | Y | Y | Perisylvian PMG；hypoplasia CC |
| 57 | p.Asn1117Lys | dimerization domain | Y | Y | Y | N | N | Y | Y | NR |
| 58 | p.Val1116Ala | dimerization domain | Y | N | Y | Y | N | Y | Y | pachygyria；PMG |
| 59 | p.Gly1132Glu | dimerization domain | N | NA | N | Y | N | Y | N |  |
| 60 | p.Val1167Glu | Neck/linker domain | N | NA | N | N | Y | N | N |  |
| 61 | p.Gln1194Arg | Neck/linker domain | N | NA | Y | N | Y | N | N |  |
| 62 | p.Phe1202Leu | Neck/linker domain | N | NA | N | N | Y | N | N |  |
| 63 | p.Phe1202Leu | Neck/linker domain | Y | Y | Y | N | N | Y | Y | Perisylvian PMG |
| 64 | p.Leu1336Phe | Neck/linker domain | N | NA | N | N | N | Y | N |  |
| 65 | p.His1412Tyr | Neck/linker domain | N | NA | Y | N | N | N | N |  |
| 66 | p.Leu1420Arg | Neck/linker domain | N | NA | N | N | Y | N | N |  |
| 67 | p.Pro1511Leu | Neck/linker domain | N | NA | N | Y | N | N | N |  |
| 68 | p.Glu1518Lys | Neck/linker domain | Y | N | Y | N | N | Y | Y | Pachygyria |
| 69 | p.Trp1537Arg | Neck/linker domain | N |  | Y | N | N | N | N |  |
| 70 | p.Glu1564Val | Neck/linker domain | Y | Y | Y | N | N | Y | Y | Posterior predominant pachygyria |
| 71 | p.Arg1567Gln | Neck/linker domain | N | NA | Y | N | N | Y | Y | pachygyria |
| 72 | p.Arg1567Gln | Neck/linker domain | Y | NR | Y | N | N | Y | Y | Frontal PMG |
| 73 | p.Arg1603Thr | Neck/linker domain | Y |  | Y | Y | N | Y | Y | Pachygyria |
| 74 | p.Arg1603Thr | Neck/linker domain | Y | Y | Y | Y | N | Y | Y | NR |
| 75 | p.Arg1623Gln | Neck/linker domain | Y | Y | Y | N | N | Y | Y | Anterior predominant Pachygyria |
| 76 | p.Val1750Met | Neck/linker domain | N | NA | N | N | Y | N | N |  |
| 77 | p.Arg1887His | AAA1 | N | NA | N | N | Y | N | N |  |
| 78 | p.Arg1962Cys | AAA1 | Y | Y | Y | N | N | Y | Y | Posterior predominant pachygyria |
| 79 | p.Arg1962Cys | AAA1 | Y | Y | Y | N | N | Y | Y | Focal pachygyria |
| 80 | p.Arg1962his | AAA1 | Y | Y | Y | N | N | Y | Y | Pachygyria |
| 81 | p.Arg1962Cys | AAA1 | Y | Y | Y | N | N | Y | Y | Posterior predominant pachygyria |
| 82 | p.Arg1962Cys | AAA1 | Y | Y | Y | N | N | Y | Y | Posterior predominant pachygyria |
| 83 | p.Glu2294Lys | AAA2 | Y | N | Y | N | N | Y | Y | Anterior predominant pachygyria |
| 84 | p.Arg2332Cys | AAA2 | Y | N | Y | N | N | Y | Y | Pachygyria；Hypoplasia CC |
| 85 | p.Arg2332Cys | AAA2 | N | NA | Y | N | N | Y | Y | Frontal PMG |
| 86 | p.Lys2401Asn | AAA2 | N | NA | N | N | Y | N | N |  |
| 87 | p.Lys2401Asn | AAA2 | N | NA | N | N | Y | N | N |  |
| 88 | p.Lys2401Asn | AAA2 | N | NA | N | N | Y | N | N |  |
| 89 | p.Gly2598Val | AAA3 | Y | NR | Y | N | N | Y | Y | Pachygyria；Hypoplasia CC |
| 90 | p.Glu2616Lys | AAA3 | N | NA | Y | Y | N | N | N |  |
| 91 | p.Tyr2641Cys | AAA3 | N | NA | N | Y | N | N | N |  |
| 92 | p.Thr2745Lys | AAA3 | N | NA | Y | N | N | Y | N |  |
| 93 | p.Glu2814Asp | motor dom (between AAA3-AAA4) | N | NA | N | N | Y | N | N |  |
| 94 | p.Asn3014Ser | AAA4 | N | NA | Y | Y | N | Y | Y | NR |
| 95 | p.Met3043Thr | AAA4 | Y | Y | Y | N | N | Y | Y | Bilateral temporal pachygyria |
| 96 | p.Val3065Met | AAA4 | N | NA | Y | Y | N | Y | Y | NR |
| 97 | p.Pro3173Arg | AAA4 | Y | Y | Y | Y | N | Y | Y | Pachygyria;PMG |
| 98 | p.Lys3241Thr | Stalk domain | Y | N | N | N | N | Y | Y | Posterior predominant pachygyria |
| 99 | p.Lys3241Thr | Stalk domain | Y | N | Y | N | N | Y | Y | Posterior predominant pachygyria |
| 100 | p.Lys3241Thr | Stalk domain | Y | N | N | N | N | Y | Y | Posterior predominant pachygyria |
| 101 | p.Lys3318Asn | Stalk domain | Y | Y | Y | N | N | Y | Y | Posterior predominant pachygyria |
| 102 | p.Lys3336Asn | Stalk domain | Y | Y | Y | N | N | Y | Y | Posterior predominant pachygyria；Frontal PMG;hypoplasia CC |
| 103 | p.Arg3344Gln | Stalk domain | N | NA | Y | N | N | Y | Y | Posterior predominant pachygyria; |
| 104 | p.Arg3344Gln | Stalk domain | Y | Y | Y | N | N | Y | Y | Posterior predominant pachygyria |
| 105 | p.Arg3344Gln | Stalk domain | Y | Y | Y | N | N | Y | Y | Posterior predominant pachygyria |
| 106 | p.Arg3344Gln | Stalk domain | Y | NR | Y | N | N | Y | Y | Posterior predominant agyria; hypoplasia CC |
| 107 | p.Arg3344Trp | Stalk domain | Y | Y | Y | N | N | Y | Y | Posterior predominant dysgyria |
| 108 | p.Glu3345Lys | Stalk domain | N | NA | Y | N | N | Y | Y | NR |
| 109 | p.Arg3384Gln | Stalk domain | Y | Y | Y | N | N | Y | Y | Posterior predominant pachygyria； Frontal PMG; hypoplasia CC |
| 110 | p.Arg3384Gln | Stalk domain | Y | Y | Y | N | N | Y | Y | Pachygyria |
| 111 | p.Met3438Val | Stalk domain | Y | Y | Y | N | N | Y | Y | Dysgyria |
| 112 | p.Met3405Leu | Stalk domain | Y | Y | Y | N | N | Y | Y | Posterior predominant pachygyria; hypoplasia CC |
| 113 | p.Met3405Leu | Stalk domain | Y | Y | Y | N | N | Y | Y | Posterior predominant pachygyria; |
| 114 | p.Arg3474Trp | Stalk domain | N | NA | Y | N | N | Y | Y | Anterior predominant pachygyria; hypoplasia CC |
| 115 | p.Leu3478Phe | Stalk domain | Y | NR | Y | Y | N | Y | N |  |
| 116 | p.Arg3525Cys | between stalk domain and AAA5 domain | Y | NR | Y | N | N | Y | Y | Anterior predominant pachygyria |
| 117 | p.Gly3630Ser | AAA5 | Y | Y | Y | N | N | Y | Y | Posterior predominant pachygyria；hypoplasia CC |
| 118 | p.Gly3658Glu | AAA5 | Y | NR | Y | N | N | Y | N |  |
| 119 | p.Gly3658Glu | AAA5 | Y | Y | Y | N | N | Y | Y | Anterior predominant Pachygyria；heterotopia |
| 120 | p.Ser3672Leu | AAA5 | Y | Y | Y | N | N | Y | Y | NR |
| 121 | p.Ser3672Leu | AAA5 | Y | N | Y | N | N | Y | Y | NR |
| 122 | p.Val3699Ile | AAA5 | Y | N | Y | N | N | Y | Y | Pachygyria；Hypoplasia CC |
| 123 | p.Arg3728Pro | AAA5 | N | NA | Y | N | N | Y | Y | dysgyria；hypoplasia CC |
| 124 | p.Glu3771Lys | AAA5 | Y | Y | Y | N | N | Y | Y | Posterior predominant pachygyria |
| 125 | p.His3822Pro | between AAA5 domain and AAA6 domain | N | NA | Y | N | N | Y | Y | Focal cortical dysplasia |
| 126 | p.Pro3942Ser | between AAA5 domain and AAA6 domain | N | NA | Y | N | N | Y | Y | Cerebellar hypoplasia |
| 127 | p.Arg3989Cys | between AAA5 domain and AAA6 domain | N | NA | N | N | Y | N | N |  |
| 128 | p.Leu4179Ser | AAA6 | Y | N | Y | N | N | N | N |  |
| 129 | p.Arg4449Pro | AAA7 | Y | Y | Y | N | N | Y | Y | NR |
|  | | | | | | | | | | |
|  |  |  |  |  |  |  |  |  |  |  |
